# Modelling Spatial and Temporal Forest Cover Change Patterns (1973-2020): A Case Study from South Western Ghats (India)

**DOI:** 10.3390/s8106132

**Published:** 2008-10-01

**Authors:** Amarnath Giriraj, Mohammed Irfan-Ullah, Manchi Sri Ramachandra Murthy, Carl Beierkuhnlein

**Affiliations:** 1 Forestry and Ecology Division, National Remote Sensing Centre, Hyderabad-500 037, India; E-Mail: murthy_msr@nrsa.gov.in; 2 Department of Biogeography, University of Bayreuth, Bayreuth D-95447, Germany; E-mail: carl.beierkuhnlein@uni-bayreuth.de; 3 Forestry, Ecology and Natural Resources, RMSI Private Limited, NOIDA, UP, India; E-mail: irfan26@gmail.com

**Keywords:** Forest cover change, tropical forest, GEOMOD, monitoring, Western Ghats

## Abstract

This study used time series remote sensing data from 1973, 1990 and 2004 to assess spatial forest cover change patterns in the Kalakad-Mundanthurai Tiger Reserve (KMTR), South Western Ghats (India). Analysis of forest cover changes and its causes are the most challenging areas of landscape ecology, especially due to the absence of temporal ground data and comparable space platform based data. Comparing remotely sensed data from three different sources with sensors having different spatial and spectral resolution presented a technical challenge. Quantitative change analysis over a long period provided a valuable insight into forest cover dynamics in this area. Time-series maps were combined within a geographical information system (GIS) with biotic and abiotic factors for modelling its future change. The land-cover change has been modelled using GEOMOD and predicted for year 2020 using the current disturbance scenario. Comparison of the forest change maps over the 31-year period shows that evergreen forest being degraded (16%) primarily in the form of selective logging and clear felling to raise plantations of coffee, tea and cardamom. The natural disturbances such as forest fire, wildlife grazing, invasions after clearance and soil erosion induced by anthropogenic pressure over the decades are the reasons of forest cover change in KMTR. The study demonstrates the role of remote sensing and GIS in monitoring of large-coverage of forest area continuously for a given region over time more precisely and in cost-effective manner which will be ideal for conservation planning and prioritization.

## 1. Introduction

The value of forests to the world's human population is becoming increasingly evident. Excessive alterations of the global environment by the human activities have led to various changes in the global biogeochemical cycles, transformation of land and have increased the mobility of many biota. These anthropogenically-induced changes have triggered the sixth major extinction event in the history of life on this earth and have caused widespread changes in the global distribution of organisms [1-3]. With increase in resource requirement, more and more natural area are encroached by humans for resource exploitation leading to the loss of biological diversity, which are potential resources for the future evolution.

Tropical forests, although covering less than 10% of the land areas, represent the largest terrestrial reservoir of biological diversity, from the gene to the habitat level. For example, more than 50% of known plant species grow in the tropical forests. With ongoing impacts (deforestation, degradation, fragmentation, and local extinction) tropical forests suffer from rapid land use changes [4]. Agents of deforestation are bringing agricultural expansion, commercial logging, commercial forestry, mining, hydropower, industry, urbanization, road building and biotic pressures inside the forest ecosystems. Studies [1, 5] have suggested that land use changes are likely to have a greater impact on biodiversity reduction than climate change, nitrogen deposition, biotic exchange or increased carbon dioxide concentration. Thus, the development of a reliable tool for protected areas (PA) is urgently needed. Further emphasis should be placed on specific vegetation types, key species, important habitats and on vulnerable landscape units. In most tropical regions, land conversion forces the decline of species abundance and range. Populations become increasingly vulnerable to collapse if exposed to additional human impact [6] such as pollution or climate change. Hence, it is necessary to address systematic conservation planning [7] for large areas to ensure the viability and long-term persistence of populations and biodiversity in situ. Biodiversity loss is related to a loss of ecosystem functioning and thereby ecological services [8, 9]. In practice, the management of reserves in the tropics is inadequately funded, unplanned and often threatened by illegal extraction of forest products or commercial activities [10]. Conservation management of naturally occurring undisturbed areas should ensure that their natural values are retained in the face of internal natural dynamics, disturbances from outside, and varied anthropogenic pressures.

The Western Ghats are one among the 34 biodiversity hotspots of the world [11]. Considering KMTR's importance for the conservation of the wider region's unique biodiversity, an assessment of forest cover and forest cover change is needed. No systematic assessment has been conducted to determine KMTR's remaining forest cover, its fragmentation patterns, or the rate at which forest cover is changing. It is therefore necessary to determine accurately the rate and spatial patterns of these processes to formulate sustainable strategies for conservation and monitoring of relatively undisturbed landscapes. Such kind of assessments can only be produced via analysis of satellite imagery and ancillary information, because on-the-ground records of impacts from agricultural conversions and logging are either inaccessible or non-existent. Satellite remote sensing offers consistent observation of tropical forests cover dynamics at a fine scale [12] with more precision and in a cost-effective manner. Thus detection of land cover change both at a spatial and temporal scale using satellite images such as Landsat, IRS, SPOT is one of the most valuable contributions to natural resource management and biodiversity assessment [13-15]. Analysis of multi-temporal satellite data offers accurate estimation of forest cover and deforestation rates [12, 14-15].

The objective of this study is to characterize spatial and temporal patterns of forest cover change, its rate of deforestation, and to develop future scenarios using remotely sensed data in a case study area of Kalakad-Mundanthurai Tiger Reserve, South Western Ghats, India. The research also focuses in identifying remnant intact patches of evergreen forest using multi-temporal satellite data (1973 – 2004) for its future scenario. These inputs are useful to delineate potential conservation areas and monitoring parameters in one of the ecologically most sensitive biodiversity hotspots of the earth.

## 2. Study area

**Figure 1.**
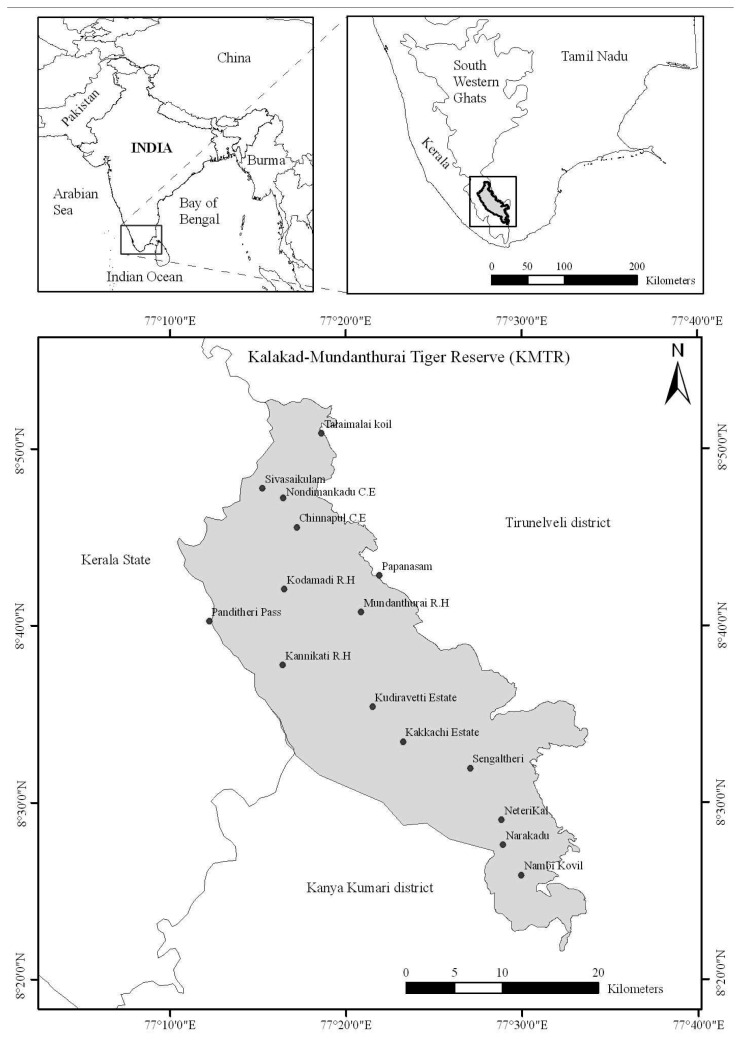
Geographic position of the Kalakad–Mundanthurai Tiger Reserve (KMTR), South Western Ghats, located in the Tirunelveli and Kanykumari districts of Tamil Nadu, (India).

The Kalakad-Mundanthurai Tiger Reserve (KMTR) is located in the southern end of the Western Ghats (hereafter “WG”), Tamil Nadu (India) (Figure 1) and lies between 8°21′ - 8°52′ N latitude and 77°10′ - 77°33′ E longitude in the biogeographic provinces [16] 4.1.1 (Malabar rainforest) and 4.14.4 (Deccan thorn forest). The area falls in two districts, namely Tirunelveli and Kanya Kumari of Tamil Nadu and is bound on the west by Kerala State. KMTR covers an area of 907 km^2^, with hills towering to majestic heights ranging from 100 to 1,880 m (Agasthiar peak). The Agasthyamalai hills in the Southern end of the WG are known for high species diversity, harbouring 2000 flowering plant species with 7.5% endemism [17]. The mid-elevation zone (700 – 1,400 m) is the tropical wet evergreen forest of the Cullenia–Mesua–Palaquium series [18]. The climate of the area typically has a minimum rainfall of 1,200 mm to a maximum of 5,000 mm. Annual average temperatures range from 13.5°C in the evergreen to 23°C in the deciduous forests. Dry period ranges from 3–5 months and number of rainy days is in the order of 89–92 days [19]. Forest types such as tropical evergreen, tropical semi-evergreen, tropical moist deciduous, tropical dry deciduous, grasslands and secondary succession exist in the study area.

## 3. Materials and Method

### 3.1. Satellite data classification

**Figure 2.**
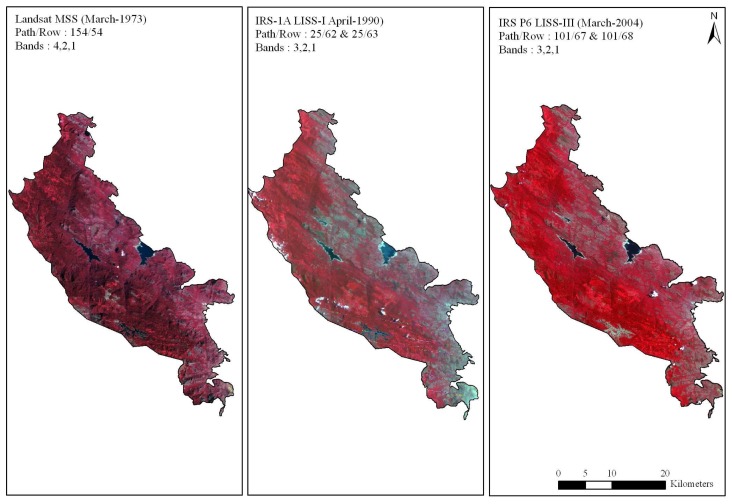
False color composites for the study region between 1973 to 2004 showing different vegetation formations, and also the variation in tone and texture in the South Western Ghats, Tamil Nadu, India.

Cloud free data of LANDSAT Multispectral Scanner (MSS) of March 1973 covering path and row 154/54 was obtained from USGS, EROS Data Center, Sioux Falls, SD, IRS-1A LISS I satellite data of April 1990 and IRS-P6 LISS III satellite data of March 2004 covering path and rows viz. 101/67 and 101/68 from National Remote Sensing Agency, Hyderabad (Figure 2). LANDSAT-MSS data with a spatial resolution of 80m and the spectral wavelength (B1 0.5 – 0.6, B2 0.6 – 0.7, B3 0.7 – 0.8 and B4 0.8 – 1.1 μm), IRS-1A LISS I with a spatial resolution of 72.5m and the spectral wavelength (B1 0.45 -0.52, B2 0.52 - 0.59, B3 0.62 - 0.68 and B4 0.77 - 0.86μm) and IRS-P6 LISS-III with a spatial resolution of 24m and the spectral wavelength of four bands (B2 0.52 – 0.59, B3 0.62 – 0.68, B4 0.77 – 0.86 and B5 1.55 – 1.70μm) were analyzed in the study. Prior to geometric correction satellite images were radiometrically corrected for subsequent analysis.

**Figure 3.**
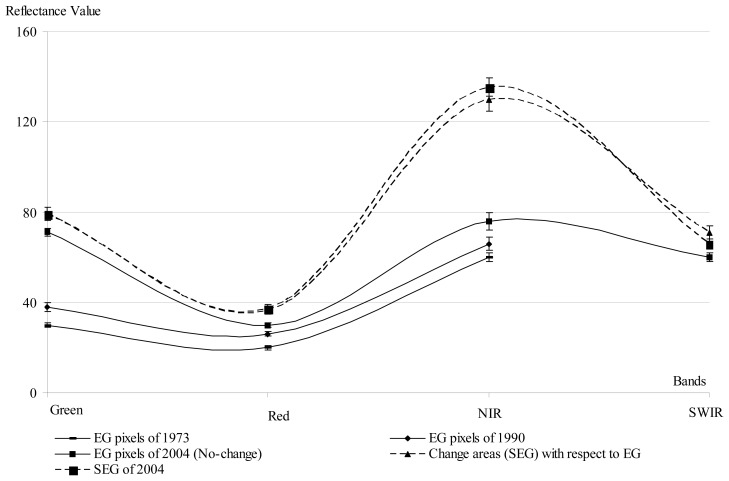
Spectral properties (X-axis has spectral bands and Y-axis has reflectance value) differentiating evergreen and semi-evergreen forest types of KMTR, South Western Ghats of Tamil Nadu, India.

The initial vegetation type map of 2004 (LISS-III) was characterized using supervised classification technique based on the information of terrain, topography and species database collected during landscape-biodiversity characterization program for Western Ghats [20]. Based on the 2004 vegetation type map inputs, an area of interest (AOI) of evergreen patches in 1973 was selected using MSS data assuming that these patches had remained unchanged from 1973 till 2004. The reflectance properties has similar trends in the evergreen and semi-evergreen patches for both the satellite images (Figure 3).

Taking the above criteria into consideration 1973 MSS image and 2004 LISS-III image were used to generate the LULC maps of 1973 and 2004 respectively. Likewise all the spectral classes were assigned training sets from the geometrically corrected images and were then classified. The maximum likelihood algorithm was used to classify these scenes [21]. Major forest types delineated were viz. tropical evergreen, semi-evergreen, moist deciduous, dry deciduous, dry evergreen, grasslands, scrubs, reeds (Ochlandra sp.) and orchards. The tone and textural differences in these forest types can be clearly seen in satellite imagery (Figure 2). The classified vegetation map was validated by verification on ground and found to be 85% accurate. Finally, IRS LISS-III dataset were re-sampled to 80m (equivalent to MSS) to facilitate comparison.

### 3.2. Predictive modelling of evergreen forest

**Figure 4.**
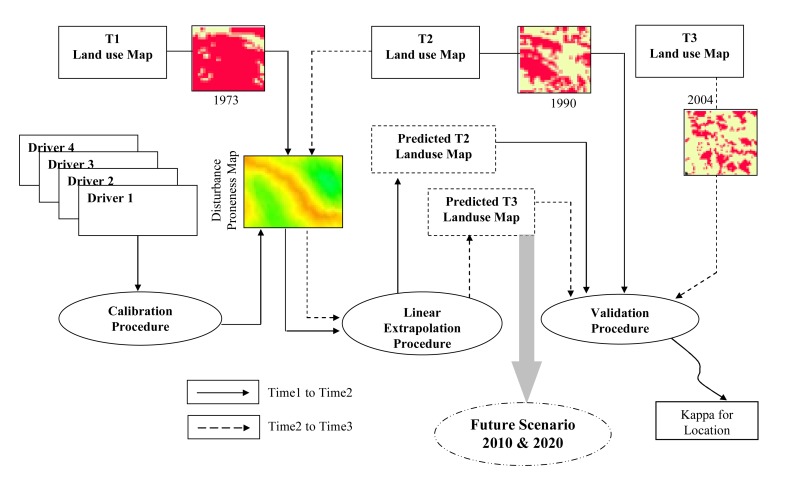
GEOMOD based modelling of evergreen forest change using spatial drivers, extrapolation and validation procedure for the future evergreen forest scenario in KMTR.

An approach which combines time series evergreen forest areas change between 1973 and 2004 and future change of its location and quantity was modelled within the geographical modelling (GEOMOD) framework (Figure 4). GEOMOD is a simple unidirectional linear change modelling tool [22] that uses suitability image/s, produced by combining a variety of driver images to predict locations of change for given quantity of change between two time periods. User re-runs GEOMOD trying to obtain higher and higher values of Kappa. Once user decides to accept a result, GEOMOD can use the “suitability” map and the selected weights to model the land cover of a future year based on only attributes. The most interesting part of this type of change modelling is in its ability to model location specific change for different quantities of change.

GEOMOD can evaluate change in two land-use types at a time. Therefore, each of the vegetation and land-cover map was reclassified as evergreen (evergreen) and non-evergreen (semi-evergreen, deciduous and other land-cover) areas. The most common reclassification is to classify all undisturbed forest as type 1, and all other land-use types as type 2, which can be categorised as area having human intervention e.g. abandoned evergreen areas, orchards, reeds (*Ochlandra* spec.). Area estimation is done using reclassified image to know how many forest pixels existed for the particular time period. Future rate of change is calculated using the simple subtraction to find the area deforested during interim period.

**Figure 5.**
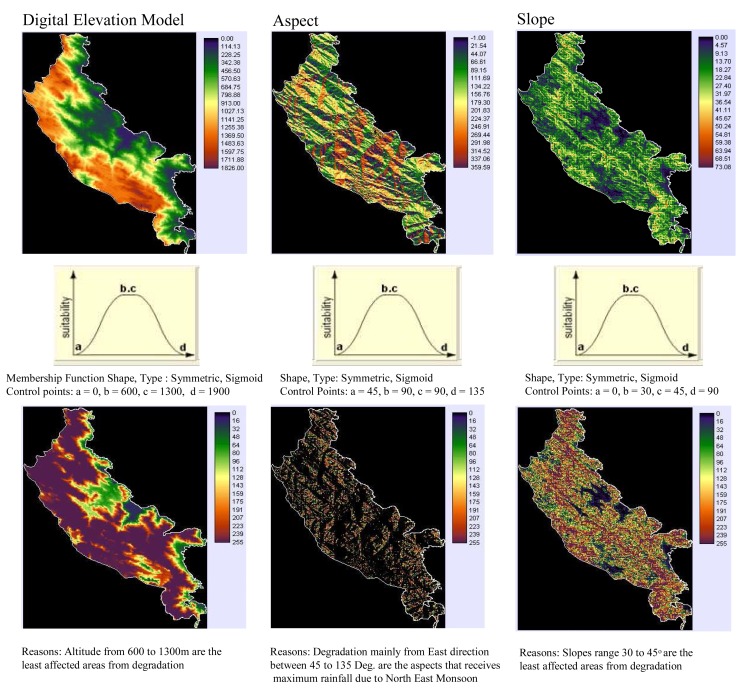

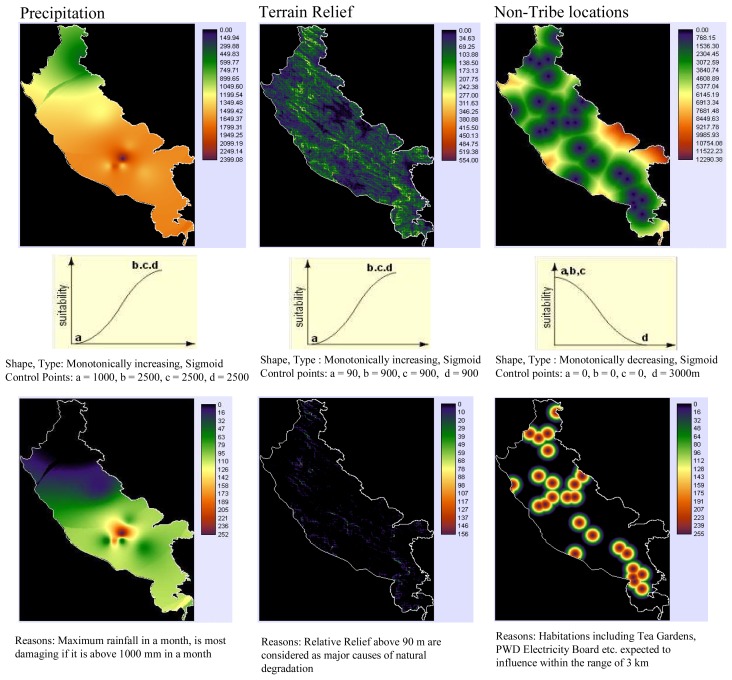

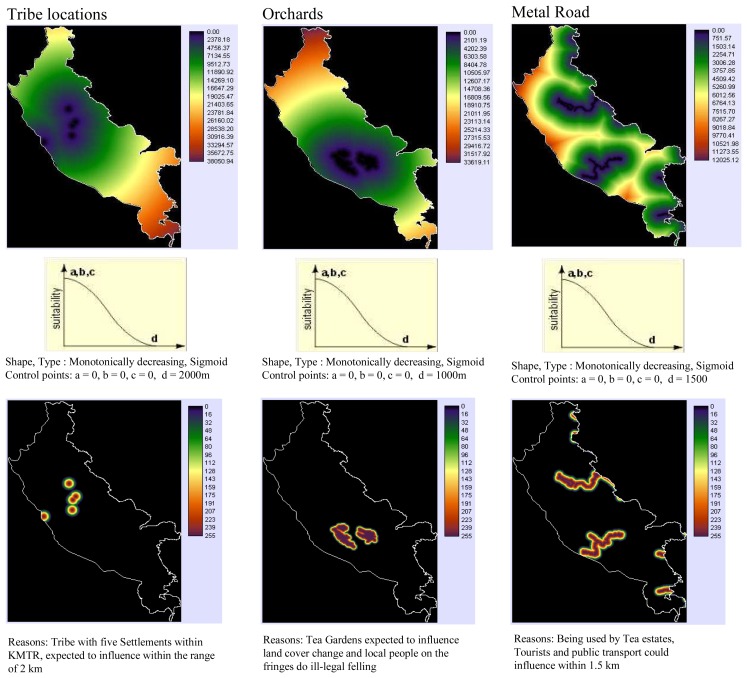

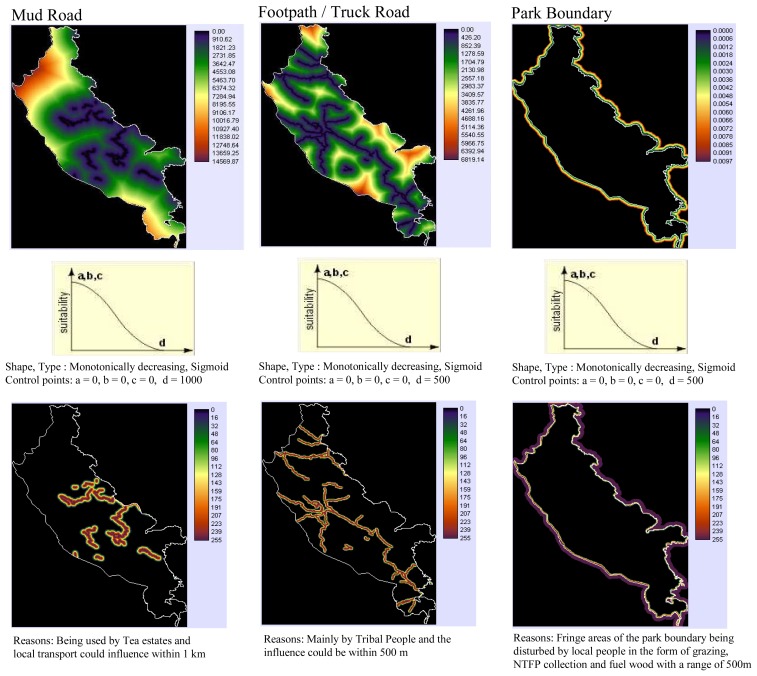

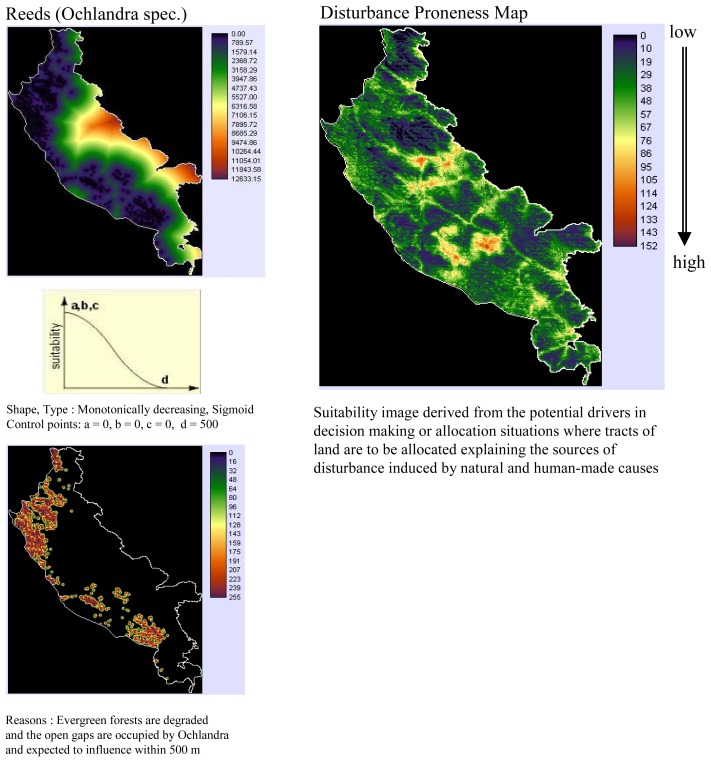
**(a)** List of potential spatial drivers (DEM, aspect, slope) prepared using multi-criteria decision support to generate suitability image for the evergreen forest change modelling of KMTR, South Western Ghats, Tamil Nadu, (India). **(b)** List of potential spatial drivers (precipitation, terrain relief, locations of non-tribe) generated using multi-criteria decision support to generate suitability image for the evergreen forest change modelling of KMTR, South Western Ghats, Tamil Nadu, (India). **(c)** List of potential spatial drivers (locations of tribe, orchards, metal road) generated using multi-criteria decision support to generate suitability image for the evergreen forest change modelling of KMTR, South Western Ghats, Tamil Nadu, (India). **(d)** List of potential spatial drivers (mud road, footpath and park boundary) generated using multi-criteria decision support to generate suitability image for the evergreen forest change modelling of KMTR, South Western Ghats, Tamil Nadu, (India). (e) List of potential spatial drivers as reeds (*Ochlandra* spec.) and suitability map generated using IDRISI program for the evergreen forest of KMTR, South Western Ghats, Tamil Nadu, (India).

Drivers explaining the evergreen forest change (Figures 5a-d) which include altitude, slope, aspect, proximity to protected area (PA) boundary, settlement, tea and coffee plantation, road and footpath, rainfall intensity and locations of reeds (*Ochlandra* spec.) were integrated using multi-criteria decision support system with appropriate fuzzy membership functions into single suitability image [23]. This suitability image was then used in GEOMOD to model change from 1973 to 1990, 1990 to 2004 and then from 2004 to 2020. All driver maps used for each calibration run are added together to create (disturbance proneness area) suitability map (Figure 5e). GEOMOD uses this map of ranked potentials, or likelihoods to simulate deforestation at a third point in time, the results of which can be validated against the actual map of that same time period to test how well the drivers did in predicting the spatial pattern of deforestation. This ‘test’ is called the validation process and is discussed further below.

### 3.3. Validation

To validate the results created by GEOMOD, the actual evergreen map at a known point in time is compared with the predicted evergreen map of that same time based on analysis of the pattern at an earlier point in time using “Validation” tool available in IDRISI module. In the past, measures of the ‘goodness of fit’ commonly were performed by using a simple percent correct measure or, at best, a multiple-resolution percent correct [24-25] but this provides little assessment of a model's ability to predict the correct quantity of change versus its ability to identify the correct location of change [22, 26]. Spatial measures of ‘goodness of fit’ have been developed that measures the degree to which a simulated map agrees with a reality map with respect to both location (Kappa-for-location) and quantity of correct cells (Kappa-for-quantity).

## 4. Results and Discussion

### 4.1. Vegetation types

**Figure 6.**
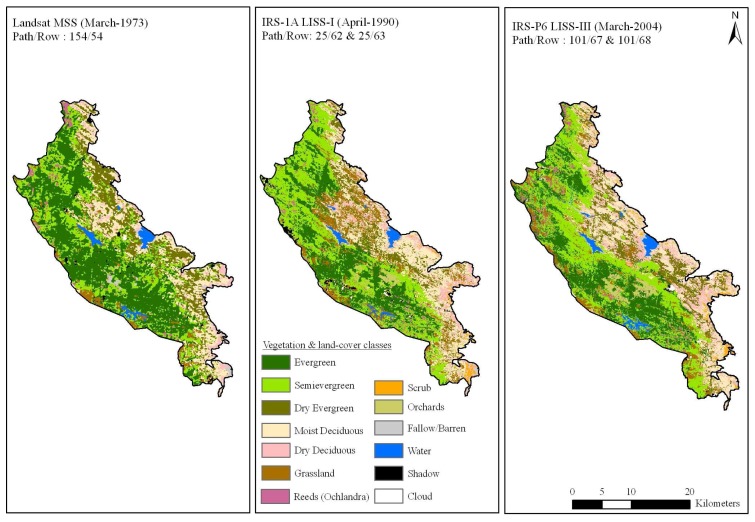
Vegetation and land cover classification map for the study region (1973 & 2004) of KMTR, South Western Ghats, Tamil Nadu, (India).

The changes in the vegetation and land cover based on digital classification of satellite data shows a significant decrease of evergreen forest mostly to semi-evergreen forest types (Table 1). Of the total area covered by natural vegetation (857 km^2^) the evergreen and semi-evergreen forest occupied 60% of the area. Before finalizing the vegetation and land-cover map, it was thoroughly checked in the field with GPS points. The overall accuracy stands at 72% with a kappa statistics of 0.70. Using this data, a final multi-temporal vegetation and land-cover classification map was brought out (Figure 6). In 1973, evergreen forest constituted 316 km^2^, followed by semi-evergreen having 194 km^2^. In 2004 the evergreen forest has degraded to 188 km^2^ (i.e. 40% loss of land-cover where as semi-evergreen forest gained by 36% to 265.1 km^2^). Grassland covering 73 km^2^ is largely distributed in Kodayar, Manjamparai and adjoining areas of Agasthyamalai region. The area under grassland has shown a significant increase of approx. 166% during 1973 – 2004.

### 4.2. Land-cover change analysis

Change detection analysis was performed between two-time period (1973-1990 & 1990-2004) and change matrix is given in Table 2. We have assessed forest cover changes in two different levels, as before and after declaration of protected area. In the first case we observed large-scale changes in forest cover between 1973 and 1990 mainly from evergreen to semi-evergreen forest type (122.39 km^2^) which constitute ca. 38% of the total land-cover changes, evergreen to reeds (*Ochlandra* spec) (3.17 km^2^), and evergreen to orchards (3.33 km^2^). Conspicuous changes in semi-evergreen forest type were to grassland (29.05 km^2^), *Ochlandra* (4.94 km^2^) and Orchards (4.67 km^2^). It is observed that the vegetation cover change is mostly in patch sizes either of ≥100 ha or 10-50 ha. In the second case (i.e. after declaration of PA in 1988) the observed changes are very less compare to the first case (i.e. before declaration of PA). Between 1990 and 2004 the loss of evergreen forests is less than 16 km^2^ and an increase of semi-evergreen forest by 29.4 km^2^. Though the area is declared as protected area, it is facing continuous disturbances and the causes behind forest cover changes are illegal timber cutting and extraction of other forest sources.

The evergreen forests of the KMTR have undergone extensive degradation during the two and half decades showing 16% of the evergreen forest being degraded primarily in the form of selective logging and clear felling to raise plantations of coffee, tea and cardamom. This has resulted in growth of secondary successional stages such as semi-evergreen out of the major phenological types. Study [27] has pointed out that there is significant loss of biological rich areas between 1960 and 1990 (85.6 km^2^ to plantation, 42.0 km^2^ to encroachment and 36.4 km^2^ to reservoirs). The present study quantified 8% increase of reeds (*Ochlandra* spec) patches since 1972 and is further continuing due to anthropogenic pressure on the intact forest which can lead to secondary succession or degradation process. Most important cause for the growth of *Ochlandra* is encroachment of cardamom and coffee plantation into evergreen forest carried out over vast areas, which include Uttu, Kakachi and Sengaltheri. Abandoned plantations undergrowth is invaded by reeds, which prevents all other regeneration as this reed is highly combustible during the dry season. Additionally recurring fires gradually enlarge the openings in the forest and is gradually replaced by reeds.

Spatial patterns of forest dynamics suggest two main processes of forest clearing, including broad-scale conversion and degradation of forests and conversion of commercial plantations (tea and coffee). Major threat to forests is not outright deforestation, rather forests and their biota are suffering from (1) simplification, where structurally rich native forests are converted to simplified secondary stands or other forest types due to harvesting of selective species [28-29], and (2) fragmentation, where remaining tracts of native forests are separated into smaller patches by anthropogenic activities, resulting in a terrain that is hostile to many species and posses barriers to movement [30-32]. This may result in significant loss of natural resources and biodiversity.

For the studies involving change estimation over a spatial and temporal domain it is important to have ground-based surveys to validate the actual changes, but such surveys are rare and in most cases do not exist in tropics [33]. Since the tropical forests are the most stressed among all the forest types and are rich in biodiversity, it is important that the changes in the tropical forest needs to monitor effectively. To analyze such databases, we need to find some indirect validation methods for the past satellite derived data [33]. The comparative evaluation of spectral properties (NIR, SWIR bands) along with species composition data in the change and no-change area could be good indirect evidence and help in demarcating the distinct vegetation types, which have undergone changes over three decades. This approach provides a reliable means of monitoring the landscape level transformation over a temporal frame [33].

Mainly forest is lost to areas that are closer to the settlement, roads and plantations; however it is observed that the reed growth (*Ochlandra* spec) is more prolific in the vicinity of the settlements and roads. It has also been observed that the rainfall intensity has significant impact over the natural degradation of the forests in the east facing slopes where the intensity of erosion and runoff is more due to heavy downpour.

### 4.3. Modelling evergreen forest cover change

Multi-criteria evaluation (MCE) a decision support tool was used to generate suitability image to identify evergreen forest areas prone to change. Fuzzy based membership function with its shape and type was used to detect the pixels which are suitable and not-suitable for change. For e.g., DEM which uses membership function as symmetric and type as sigmoid where the altitude between 600 to 1,300 m are identified as the least degraded areas. Ground information was randomly collected for all the potential drivers used in this study as an input source for multi-criteria decision wizard. Time series inputs along with suitability map were used in Geographic Modelling (GEOMOD) framework to predict future areas of change for its conservation planning and restoration of tropical rainforest system. Similar approaches were carried out in Bannerghatta National park (India) to identify deforestation rate and also proposed modelling as a tool for conservation of forest resources and its sustainable management [34].

**Figure 7.**
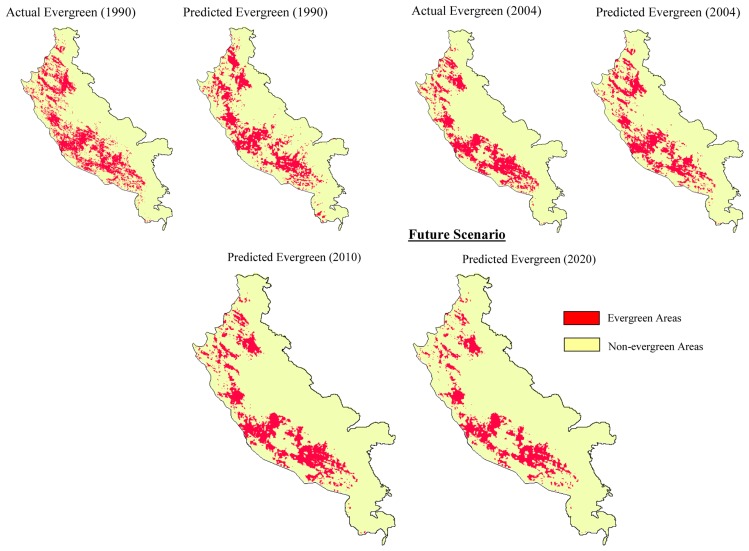
Comparison of actual and predicted evergreen forest change between 1990 & 2004 using GEOMOD modelling and also the future scenario map of 2010 & 2020 for the study region (KMTR), South Western Ghats, Tamil Nadu (India).

**Figure 8.**
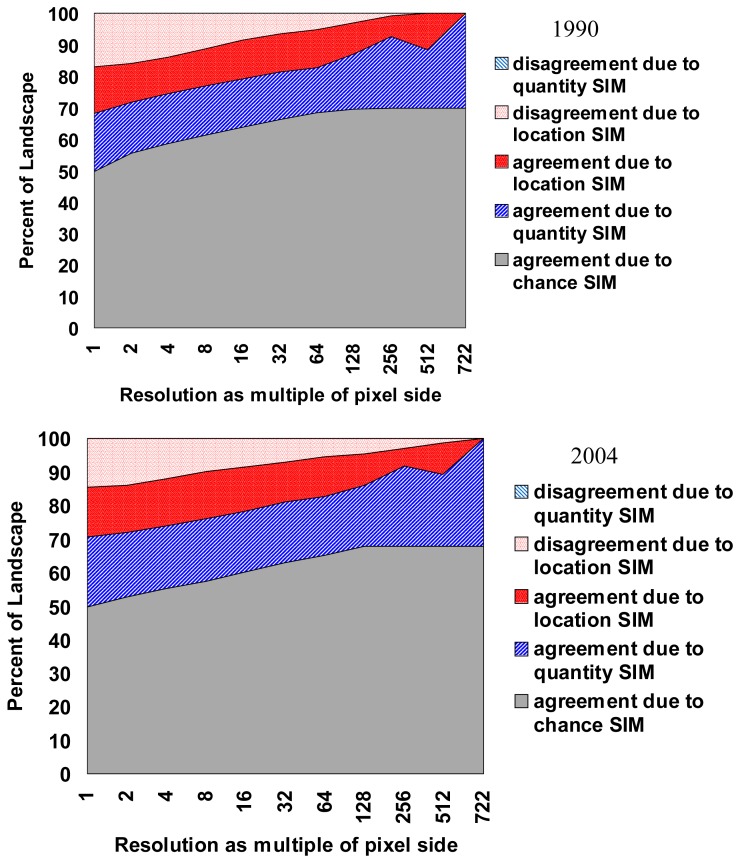
Evaluation of the evergreen forests change using Kappa validation module (IDRISI) for the actual and reference image (1990 & 2004) of KMTR, South Western Ghats, Tamil Nadu, (India).

Results indicate that between 1973 and 1990 (the protection began in 1987) about 42% of the total non-degraded forest was actually lost to the degraded category, however the rate of degradation substantially reduced after the protection became effective and only 9% of the total forest that remained in 1990 was lost to the degraded category (Figure 7 and Table 3). Using GEOMOD it was perceived that even if the same level of protection persists; an additional 27% of the non-degraded forest would be lost to the degraded category by 2020. The results were validated using validation tool of IDRISI software. The pixel location and quantity between actual and predicted map of 1990 showed 80% accuracy, while in the year 2004 it was 88% accuracy (Figure 8). Based on the validation inputs, predicted map of 2010 and 2020 was prepared (Figure 7).

Despite legal protection from major human activities, the region is subjected to various processes that ultimately prove detrimental to the sustenance of the native forest system. The focused priority on conservation of these patches may be helpful to sustain the biological diversity as these patches of evergreen forests provide unique habitats for various endemic plant species and wildlife. In this context the moderate spatial and high spectral resolution data from wide field sensors, can be used for generation of extensive information regarding vegetation area, patch shape and size, fragmentation patterns and porosity which are the major indicators of the disturbance and land-use change in a region.

The natural disturbance like fire, wildlife, reeds presence in evergreen forest and invasions and soil erosion induced by anthropogenic pressure on the forests over the decades are the reasons for changes in the overall composition and result in establishment of habitat generalist species in KMTR. Further we also intend to study evergreen patches of change and no-change areas for species richness, composition, functional and structural process to understand ecosystem functioning and behavioural pattern.

## 5. Conclusions

The study has demonstrated the utility of remote sensing and GIS in detecting forest cover change, identified magnitude and rates of deforestation. Temporal change analysis using GEOMOD has shown that an additional 27% of the non-degraded forest would be lost to the degraded category by the year 2020 in the region. The comparison map clearly explains the probable locations of degradation within the protected areas if the present trends of deforestation and practice of protection continues. Therefore areas like Agasthyamalai and Kodayar pockets should be prioritised for protection, as they are more vulnerable for degradation. We point out there should be new potential satellite sensors to assess regional scale of forest and land-cover mapping at a ‘fine’ spatial resolution (180 – 300 m) for the emerging environmental change issues. Such program not only provides better land-cover information needs at global and regional scales, but also at sub-regional and national levels. Indeed such data could establish the link between global and local observations. Further deforested pixels in the protected areas need to be monitored using high-resolution remote sensing data, ground observation explaining the positive and negative feedback from the region, which could be a useful tool for assessing productivity and enriching biodiversity. This approach can deliver accurate estimates of forest cover change, as well as long-term monitoring of ecological sensitive areas, represent keystone for conservation policy, which will be a robust, cost-effective and feasible tool in the coming decades.

## Figures and Tables

**Table 1.** Vegetation and land-cover distribution in KMTR, South Western Ghats, Tamil Nadu, India for the years 1973 and 2004 using satellite.

**S.No**	**Types**	**1973**	**1990**	**2004**
*A*	*Phenelogical types*			
1	Evergreen	316.72	182.00	166.49
2	Semi-evergreen	194.40	255.72	285.10
3	Moist deciduous	143.59	146.01	132.02
4	Dry deciduous	38.95	72.50	98.30
5	Dry evergreen	136.13	67.87	61.55
6	Grassland	27.36	114.19	75.14
	**Subtotal**	**857.16**	**838.29**	**818.60**
*B*	*Other landcover types*			
7	Shrubs	1.11	13.20	16.01
8	Ochlandra	13.56	13.80	23.74
9	Orchards	2.33	10.17	16.42
10	Fallow/barren	10.04	7.59	9.86
11	Water	15.22	13.40	16.86
12	Shadow	6.97	8.79	2.50
13	Cloud	1.07	2.21	3.46
	**Grand total**	**907.46**	**907.46**	**907.46**

**Table 2.** Change matrix for the KMTR between 1973 to 1990 and 1990 to 2004, South Western Ghats of Tamil Nadu (India).

**1990**	**1**	**2**	**3**	**4**	**5**	**6**	**7**	**8**	**9**	**10**	**Total**
**1973**											
**1**	159.33	122.39	0.26	0.02	0.00	3.17	26.79	0.00	3.33	1.43	**316.72**
**2**	21.24	115.67	9.95	7.58	0.00	4.94	29.05	0.05	4.67	1.25	**194.40**
**3**	0.00	0.00	84.07	30.63	0.00	0.21	22.49	5.16	0.00	1.03	**143.59**
**4**	0.00	0.00	17.67	14.47	0.00	0.01	1.56	4.39	0.00	0.85	**38.95**
**5**	0.00	5.00	30.08	18.77	65.77	0.17	5.16	2.03	0.00	9.16	**136.14**
**6**	0.00	6.47	0.10	0.00	0.13	5.61	0.93	0.01	0.00	0.31	**13.56**
**7**	0.00	0.00	0.00	0.00	0.00	0.54	26.04	0.19	0.01	0.58	**27.36**
**8**	0.00	0.00	0.00	0.00	0.00	0.01	0.00	0.81	0.00	0.29	**1.11**
**9**	0.00	0.00	0.00	0.00	0.00	0.00	0.00	0.07	1.11	1.15	**2.33**
**10**	1.43	6.19	3.88	1.03	1.97	2.14	3.66	0.48	1.05	11.46	**33.29**
**Total**	**182.00**	**255.72**	**146.01**	**72.50**	**67.87**	**16.80**	**115.68**	**13.20**	**10.17**	**27.51**	**907.46**

**2004**	**1**	**2**	**3**	**4**	**5**	**6**	**7**	**8**	**9**	**10**	**Total**
**1990**											
**1**	116.80	58.07	0.40	0.27	0.59	3.83	0.40	0.01	0.65	0.98	**182.00**
**2**	35.60	166.99	10.80	19.75	4.92	6.18	5.83	0.01	1.52	4.12	**255.72**
**3**	0.00	32.44	53.79	33.92	7.46	0.08	5.37	5.80	0.00	7.15	**146.01**
**4**	0.00	0.00	41.31	17.06	8.24	0.03	2.06	2.58	0.01	1.21	**72.50**
**5**	0.00	3.85	12.75	17.94	31.58	0.04	0.16	0.42	0.00	1.13	**67.87**
**6**	0.00	8.42	0.05	0.00	0.07	3.97	0.80	0.00	0.30	0.18	**13.80**
**7**	5.80	12.96	0.00	0.20	8.18	11.29	58.43	3.17	9.27	6.38	**115.68**
**8**	0.01	0.28	9.28	0.00	0.00	0.00	0.79	2.76	0.01	0.06	**13.20**
**9**	0.00	0.00	0.99	1.01	0.00	0.68	0.07	0.00	4.41	3.00	**10.17**
**10**	8.28	2.09	2.65	8.15	0.52	0.63	1.23	1.26	0.24	5.47	**30.52**
**Total**	**166.49**	**285.10**	**132.02**	**98.30**	**61.55**	**26.75**	**75.14**	**16.01**	**16.42**	**29.68**	**907.46**

1 - Evergreen; 2 - Semi-evergreen; 3 - Moist Deciduous; 4 - Dry Deciduous; 5 - Dry Evergreen; 6 – Reeds; 7 - Grassland; 8 - Scrub; 9 - Orchards; 10 – Others

**Table 3.** Current and future scenarios for the evergreen forests loss observed in KMTR of South Western Ghats, Tamil Nadu (India).

**Evergreen Forest**	**Area (sq.km)**

1973	316
1990	182
2004	166


**Current Scenario**	

**Category**	**No. of Pixels**
Evergreen forest - 1973	47709
Evergreen forest - 1990	27616
Total change	20093

Total change (Sq.km)	129
Annual rate of change (Sq km)	7.56

Evergreen forest - 2004	24903
Total change	2713

Total change (Sq.km)	17
Annual rate of change (Sq km)	1.2


**Future Scenario**	

**Change in Evergreen Forest**	**Area (Sq.km)**
2010	134.72
2020	112.79
